# Structure Formation and Coupling Reactions of Hexaphenylbenzene and Its Brominated Analog

**DOI:** 10.1002/cphc.202100049

**Published:** 2021-07-18

**Authors:** Jacob D. Teeter, Paulo S. Costa, Christoph Dobner, Mamun Sarker, Alexander Sinitskii, Axel Enders

**Affiliations:** ^1^ Department of Chemistry University of Nebraska – Lincoln 639N 12th Street Lincoln NE 68588 USA; ^2^ Department of Physics and Astronomy University of Nebraska – Lincoln 855N 16th Street Lincoln NE 68588 USA; ^3^ Physikalisches Institut Universität Bayreuth Universitätsstraße 30 95440 Bayreuth

**Keywords:** graphene nanoribbons, on-surface synthesis, scanning tunneling microscopy, self-assembly, carbon nanostructure

## Abstract

The on‐surface coupling of the prototypical precursor molecule for graphene nanoribbon synthesis, 6,11‐dibromo‐1,2,3,4‐tetraphenyltriphenylene (C_42_Br_2_H_26_, TPTP), and its non‐brominated analog hexaphenylbenzene (C_42_H_30_, HPB), was investigated on coinage metal substrates as a function of thermal treatment. For HPB, which forms non‐covalent 2D monolayers at room temperature, a thermally induced transition of the monolayer's structure could be achieved by moderate annealing, which is likely driven by π‐bond formation. It is found that the dibrominated carbon positions of TPTP do not guide the coupling if the growth occurs on a substrate at temperatures that are sufficient to initiate C−H bond activation. Instead, similar one‐dimensional molecular structures are obtained for both types of precursors, HPB and TPTP.

## Graphical abstract

**Annealing and substrate control self‐assembly**: The precursor molecule 6,11‐dibromo‐1,2,3,4‐tetraphenyltriphenylene, C_42_Br_2_H_26_, is commonly used to fabricate chevron‐type graphene nanoribbons (top) via precursor polymerization that is guided by the two Br substituents. Under certain conditions, which include the substrate choice and the annealing conditions, the self‐assembly is not guided by the Br substituents, and alternative nanostructures (center and bottom) are observed. Such structurers are also observed when the non‐brominated analog hexaphenylbenzene (C_42_H_30_) is used.

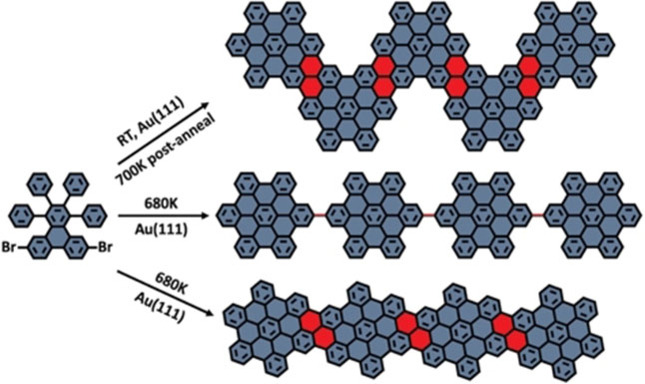

## 1 Introduction

Atomically precise graphene nanoribbons (GNRs) exhibit electronic properties, which depend strongly on their width and edge structure.^[1]^ Unlike graphene, which is a semimetal that does not have an electronic band gap and which is, by itself, generally not amenable to structural and properties design, GNRs are particularly amenable to controlled manipulation of their properties.[^2^, ^3^] Significant control parameters include the width of a GNR,^[4]^ its edge geometry,^[5]^ the edge chemistry, i. e. edge doping,[^6^, ^7^] and very recently the formation of local junctions.[^8^, ^9^, ^10^] A pioneering study, performed by Cai et.al, has demonstrated the power and versatility of the on‐surface synthesis to build tailored, atomically precise GNRs from designed precursor molecules, such as from 6,11‐dibromo‐1,2,3,4‐tetraphenyl‐triphenylene, (C_42_Br_2_H_26_),^[11]^ which we dub TPTP in this article. The procedure for on‐surface synthesis of GNRs requires the deposition of the precursor molecules on clean, crystalline substrate surfaces at or near room temperature under ultrahigh vacuum (UHV), followed by annealing of the molecules to sufficiently high temperatures to promote their polymerization and cyclodehydrogenation. A key role in this strategy is played by halogen substituents such as bromine (Br), which are located strategically upon the precursor molecules and act to guide the molecular coupling. Moderate thermal treatment of the molecules on the substrate surface (for instance at approx. 525 K for TPTP on Au(111)^[11]^ or just slightly above room temperature for TPTP on Cu(111)^[12]^ ) cleaves the carbon‐halogen bond, thus creating radical species, which diffuse across the surface where they meet to form linear polymer chains, typically via Ullmann‐like coupling. The halogen,[^13^, ^14^] as well as the substrate itself,[^12^, ^15^, ^16^, ^17^] are both parameters to control the dehalogenation temperature. This could be exploited to stimulate hierarchical coupling of precursor molecules. Once the molecules are polymerized, their originally inert molecular C−H bonds are then activated by further temperature increase (above 700 K for TPTP^11^) to form the GNR via cyclodehydrogenation reaction.

Here we explore the self‐assembly of GNR precursor molecules under conditions that deviate from the strategy above in two ways. On one hand, we compare the coupling of the prototypical chevron GNR precursor molecules used by Cai *et al*.,^[11]^ TPTP, with the structurally related but non‐brominated species hexaphenylbenzene (C_42_H_30_), dubbed HPB here. The HPB molecules form a non‐covalent 2D monolayer both on Au and on Cu surfaces, which is already known from an earlier study.^[18]^ However, we observed a thermally induced structural phase transition of those 2D layers on Cu(111) during moderate annealing, which is probably driven by intermolecular π‐bonding. On the other hand, if either molecule is deposited on a hot substrate at substrate temperatures suitable to activate the C−H bonds of the precursors, then other dendritic‐like structures are formed instead, exhibiting a bonding pattern that is in similar ways not predetermined by the Br functionalization.

## 2 Results and Discussion

The starting point for this study is HPB, which was deposited on clean Au(111) and Cu(111) surfaces with the substrates held at room temperature. Both types of substrates have been used in previous studies, which consistently found that somewhat stronger interactions of the molecules with the Cu(111) substrate result in polymer and nanoribbon formation at considerably lower temperatures, as well as GNR alignment which is directed by the principal crystallographic directions of Cu(111).[^12^, ^15^, ^16^, ^17^, ^19^]

**Figure 1:**
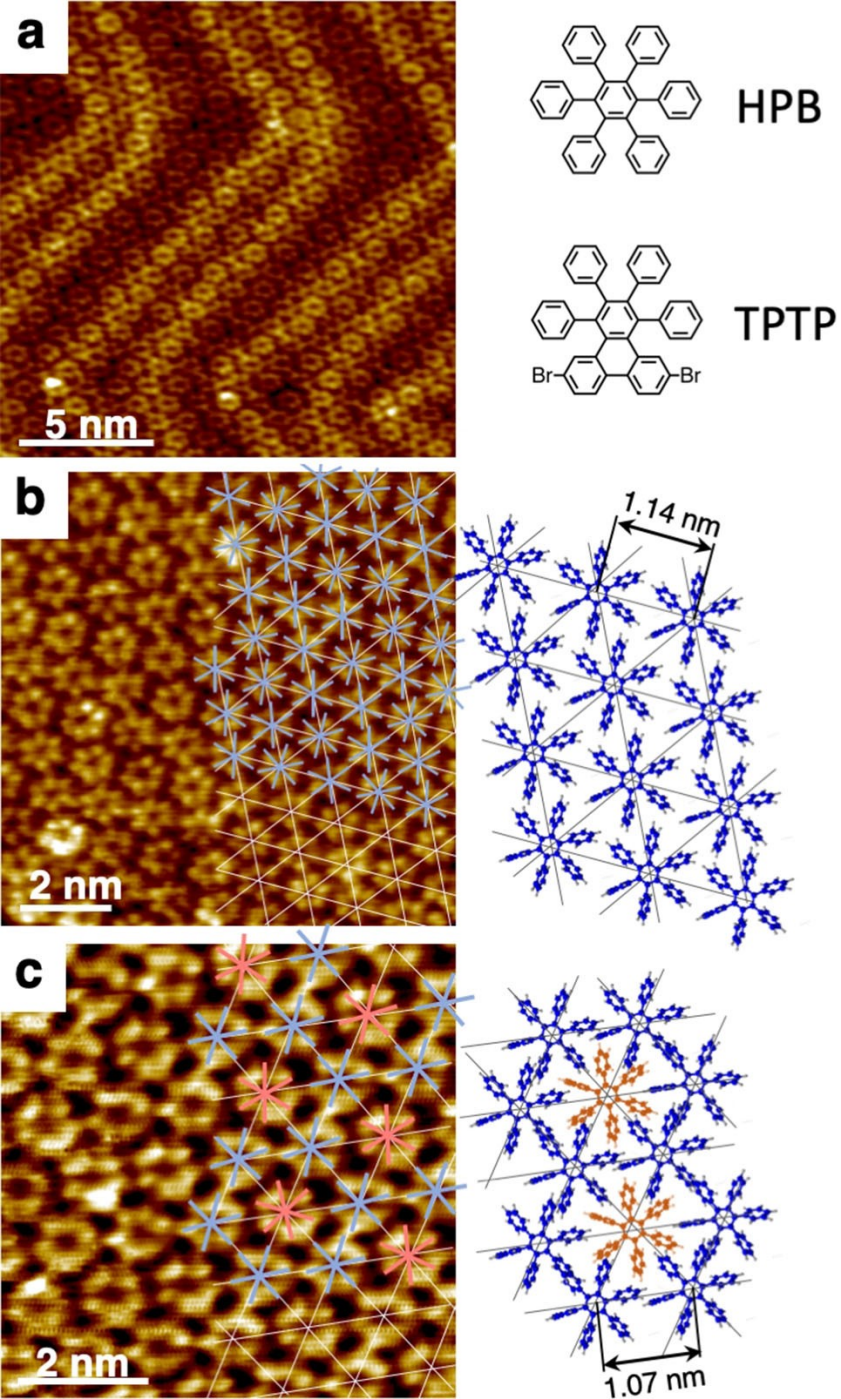
Scanning tunneling microscopy images and structure models of surface‐supported HPB monolayers. (a) HPB on Au(111), annealed to 325 K ; (b) HPB on Cu(111), annealed to 325 K; (c) HPB on Cu(111) after annealing to approx. 440 K. The white lines in (b) and (c) are aligned with the densely packed molecular rows. Colored crosses that represent the symmetry of single HPB molecules are superimposed with parts of the STM images such that they align with the phenyl rings of the HPB, which appear as bright lobes in the STM images. Actual structure models are shown to the right of each image. Red and blue colors are used to highlight particular alignments of individual molecules with respect to the molecular rows. Blue: molecular axis nearly parallel to rows; Red: molecular axis rotated approx. 20 degrees away from rows. The STM images without superimposed models are shown in the Supporting Information, for clarity.

Images acquired *via* scanning tunneling microscopy (STM) of HPB as deposited on both substrates, Au(111) and Cu(111), at room temperature followed by weak annealing to 325 K to help disperse the molecules are shown in Figures 1a and 1b, respectively. Inspection of the arrangement of molecules on both surfaces reveals that they are densely packed in very similar fashion within a single monolayer, with geometrical space filling appearing to be the driving mechanism. This implies that the lateral interaction between molecules is at least of weakly attractive nature. Sub‐molecular contrast is visible in all images in Figure 1. The molecules appear as hexagons with 6 bright lobes in the images. Following arguments presented by other authors,[^11^, ^18^] these lobes are attributed to the location of the six outer phenyl groups of the molecules, which are rotated with respect to the plane of the central benzene ring. The center‐to‐center distance of the molecules is too short for any type of C−C coupling in a hexagonal arrangement of molecules. The visual impression of “connections” between some molecules in the STM images is assumed to be an STM tip effect. In the image in Figure 1b we superimpose some of the molecules with six‐pointed asterisks, which represent the symmetry and orientation of the propeller‐like shape of the molecules, such that the axes of those asterisks match the locations of the rotated phenyl groups as well as possible. From the orientation of these asterisks and from their spacing a 2D structure model was derived, which is also shown in Figure 1b on the right. Clearly, the axes of the phenyl rings are not aligned along the molecular rows, which are represented as thin grey lines, but rather rotated away from the molecular rows by angles in the range between 0 degrees and 40 degrees, typically 20–30 degrees. As a result, the center‐to‐center spacing of the molecules along the molecular rows is 1.14 nm on average. The partial interlocking of the phenyl rings of neighboring molecules, as seen in the model, allows for dense packing of the molecules into a 2D structure in which each molecular site has the same symmetry. It is noted here that HPB films on Cu(111) have been investigated in an earlier study,^[18]^ wherein an alternative interpretation of the film structure was derived from the STM images.

Higher density packing is possible if the symmetry of the local molecular environment is reduced, as will be shown next. Post‐annealing of a HPB monolayer on Cu(111), such as the one in Figure 1b, to 440 K, triggers a rearrangement of the molecules, as seen in Figure 1c. Given that sub‐molecular contrast is still clearly visible in this image we have to conclude that neither C−H bond activation nor cyclization and planarization have occurred yet, both of which are essential steps in the formation of GNRs and which are known to eliminate sub‐molecular features in the STM images. We perform our analysis of the molecular arrangement in analogy to Figure 1b, by measuring again the rotation of the molecules’ principal axes with respect to the densely packed molecular rows. Color‐coded six‐pointed asterisks are again superimposed with the STM image. We find molecules which assume a rotation angle smaller than 5 degrees with respect to the molecular rows; those are marked with a blue asterisk. We also find molecules with considerably larger angle of rotation, of the order of 20–40 degrees, those are marked with a red asterisk. The corresponding model of the monolayer using propeller‐shaped molecule models reveals a significant change compared to the model of the as‐grown film before annealing.

Those molecules that are now aligned with their phenyl rings along the molecular rows (blue) were able to move closer together, reducing the center‐to‐center distance from 1.14 nm to 1.07 nm. Since the overlap of the phenyl groups of neighboring molecules is increased by this rearrangement, it appears that the driving mechanism is the formation of π‐bonds between those molecules. Given the chirality of the free molecules, such a π‐bond formation with all six of their respective neighbors is not possible; it would only be possible if the phenyl groups are rotated considerably around the C−C bond axis that connects them with the central benzene ring of the molecule, to about 90 degrees with respect to the molecular plane. The so‐connected molecules form a honeycomb pattern. The holes of this honeycomb structure are filled with additional molecules (red), but for them to fit into those holes they must be rotated by approx. 30 degrees with respect to the molecular rows, as shown schematically in Figure 1c. We therefore conclude that annealing of the film increases the overlap of the molecular phenyl groups and promotes the formation of stronger π‐bonds, but this requires molecular distortion, and it disfavors some other molecules, the ones in red.

**Figure 2:**
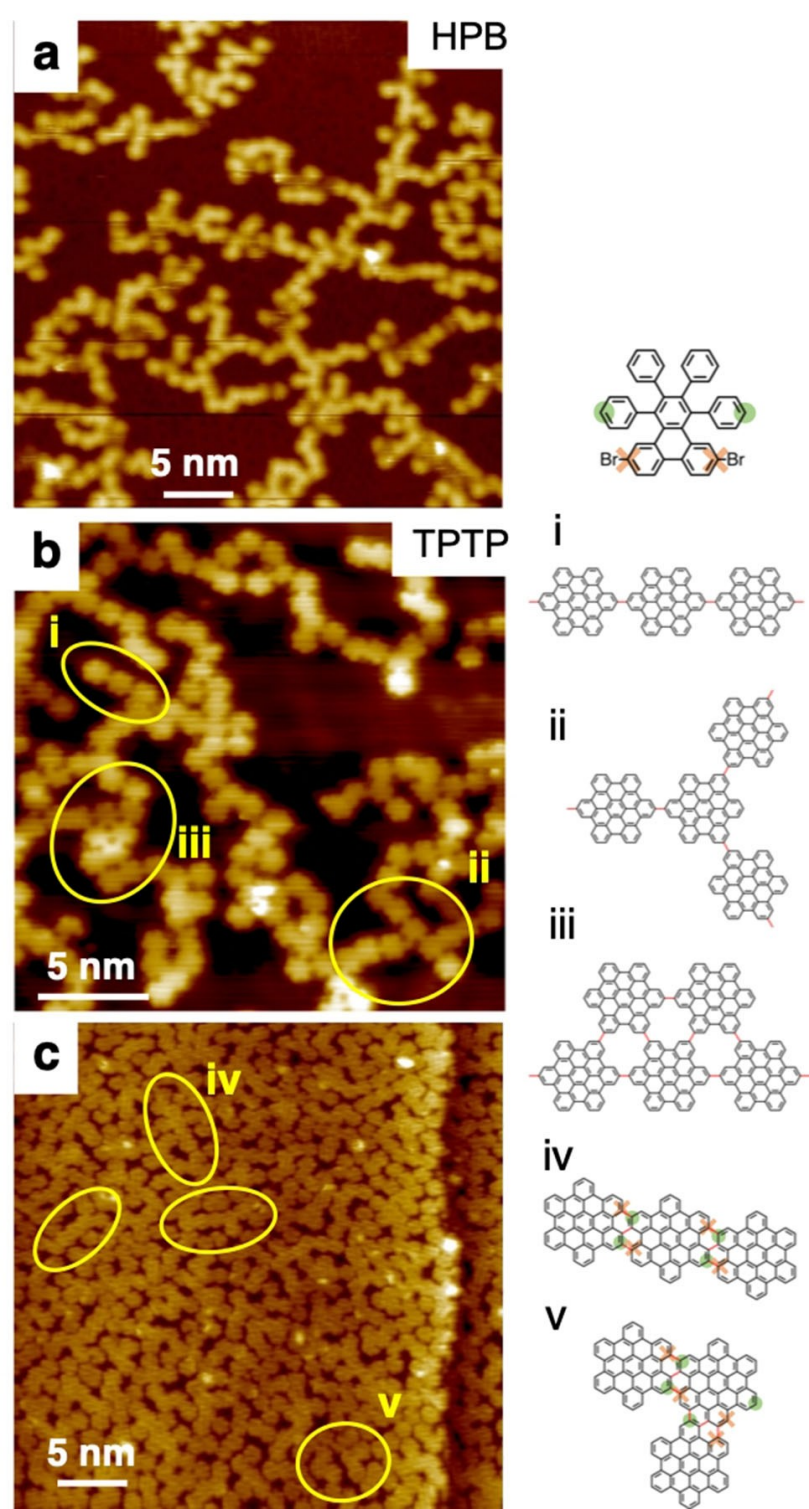
STM image of (a) HPB on Cu(111) and (b, c) TPTP on Au(111). The precursor deposition occurred at approx. 410 K substrate temperature on Cu(111) and 680 K on Au(111). (i–v) Models of structural elements observed most frequently in the STM images of TPTP in b and c, such as one‐dimensional linear chain segments (i), kinks and junctions (ii), as well as two‐dimensional arrangements (iii), are shown below the STM image. (c) high coverage of TPTP on Au(111). Single molecules, all structural elements (i–iii) and new linear (iv) and kinked (v) structure wherein the precursors are connected differently, are found in this STM image. Intermolecular covalent bonds are colored red. Possible TPTP's debrominative coupling sites (red crosses) and select dehydrogenative coupling sites (green circles) are color coded in models iv and v, in accordance with the color scheme shown in the top right panel.

The growth kinetics plays a significant a role in the molecular self‐assembly, too. To demonstrate this, we compare in the following our previously discussed approach, which is near‐room‐temperature deposition followed by post‐annealing, with an alternative approach, where the molecules were deposited from the vapor phase directly onto a hot substrate, i. e. onto the substrate held at the temperature needed for cyclodehydrogenation. An STM image of resulting molecular structures of HPB, grown on Cu(111) at 450 K, is shown in Figure 2a. We observe now lower‐dimensional dendritic chains of molecules wherein molecules form C−C bonds with typically two neighboring molecules. All molecules appear planarized and do not show any intramolecular contrast, both of which is characteristic for fully cyclodehydrogenated molecules. The observed molecular chains (see also model i in Figure 2) are only straight over very few precursor molecules; the assemblies are determined by some randomness regarding the attachment points where neighboring molecules connect. Thus, kinks within the chains, junctions between chains (ii), and small islands (iii) are commonly observed, however, molecules with more than 3 neighbors are only rarely observed. The key point here is that these structures are thus markedly different from the 2D monolayers in Figure 1, wherein each molecule is always coordinated with 6 neighbors. These structures remain unchanged at annealing temperatures of up to 550 K, it is reasonable to assume that these structures are covalently bound through C−C coupling.

What is very interesting is that very similar structures can be obtained even if the brominated TPTP precursor was used instead of the non‐brominated HPB precursor. Structures formed from TPTP by deposition, for example here on the hot Au(111), can be seen in the STM image in Figure 2b. The STM image shows the cyclized and planarized precursor molecules very clearly. Also here, straight and kinked chains, junctions, and smaller islands were formed, which is analogous to the structures from HPB in Figure 2a but distinctly different from the polymer chains leading to the chevron GNRs, which these precursor molecules usually form with the established GNR synthesis approach.[^11^, ^12^] Obviously, halogen functionalization plays no significant role in the on‐surface structure formation from TPTP under the conditions described, which is analogous to findings in a related study of 9,9’‐biantrhyl.^[17]^ Notably, the similarity of the HPB and TPTB structures suggests that here the debrominated C radical does not cause regioselectivity for dehydrogenated C−C coupling, i. e. the specific activation of a single, selected C−H bond in the presence of others. This is in stark contrast to related studies where specific activation of a select C−H bond was achieved with debrominated carbon positions.[^20^, ^21^, ^22^] At higher coverages, such as approx. 80 % of full monolayer coverage, other low‐dimensional bond patterns than those in Figure 2b are observed, see Figure 2c. New straight and kinked structures wherein the molecules are fused together at their hexagonal sides (models iv and v) are now frequently observed. Such structures require formation of as many as 3 covalent C−C bonds between any two neighbors, while at the same time allowing for denser packing of the molecules. For those structures, such as iv and v in Figure 2, it is very conceivable that structure formation was guided, at least to some extent, by the concerted action of debrominated and dehydrogenated carbon positions, as depicted in iv and v using the color code established in the top right panel in Figure 2. However, this bonding scheme using debrominated C positions cannot be consistently applied throughout those structures and thus might be coincidental.

## 3 Conclusions

The presented results are closely related to studies with established synthesis strategies of GNRs from TPTP precursors, and they explore a broader parameter space that includes halogen type, temperature and substrate material, which all determine the on‐surface synthesis of GNRs. We demonstrated that non‐covalent 2D structures are formed if the precursor molecules are not equipped with halogen functionalities. This, by itself, is not very surprising and has been shown in previous studies. What is new here is that these networks show an interesting structural phase change during moderate annealing, most likely driven by the formation of π‐bonds within the network and possibly molecular conformational changes. At considerably higher temperatures, at or near the temperature where cyclodehydrogenation usually occurs, both the non‐brominated HPB as well as the brominated TPTP species form lower‐dimensional molecular chains wherein molecules are connected via C−C bonds. The overall bond pattern is similar for HPB and TPTP in that it is not determined by the Br substituents in case of TPTP and is most likely determined by the kinetics of the reaction, which appears to be influenced also by the surface coverage. However, for TPTP we find some structures that are at least partly determined by the brominated carbon positions.

Interestingly, for HPB we obtained two distinct products on Cu(111) via two different preparation protocols at similar final substrate temperatures. A possible explanation is that the molecules, via the preparation protocol that includes room temperature deposition and post annealing, are so densely packed on the surface and interlocked with one another so that a C−C coupling reaction between neighboring molecules is geometrically hindered. By contrast, vapor deposition on a hot substrate would immediately allow C−H bond activation and C−C coupling of impinging, comparatively free molecules.

Future studies could be focused on investigating whether similar modulations in annealing conditions and the type of a substrate could affect the on‐surface coupling of other GNR precursors[^23^, ^24^] and, in particular, the precursors of modified chevron GNRs,[^14^, ^25^, ^26^] which are closely related to the molecules studied in this work.

## Experimental Section

An Omicron low‐temperature scanning tunneling microscope operated under UHV with an electrochemically etched W tip was used for all STM measurements. All STM images shown in this article were taken at 77 K sample temperature. Au(111) and Cu(111) single crystals, purchased from Princeton Scientific, were cleaned using repeated cycles of Ar^+^ ion sputtering and subsequent annealing to approx. 650 °C. Cleanliness of the crystal was checked with STM prior to molecular deposition. All sample temperatures during annealing were inferred from the heating power used, which was calibrated prior to all studies using a molybdenum test sample of identical size and shape as the single crystals, with a thermocouple attached to it. Since the actual temperature, especially at higher heating powers, depends on the heating power, time, and emissivity of the sample, the error bar of all temperatures quoted is comparatively large, and estimated to ±25 K.

Details on the synthesis and characterization of 6,11‐dibromo‐1,2,3,4‐tetraphenyltriphenylene (TPTP) can be found in our previous work.^[27]^ Hexaphenylbenzene (HPB) was acquired from Sigma‐Aldrich. TPTP and HPB molecules were deposited on Au and Cu via thermal evaporation under UHV at a pressure less than 1×10^−9^ mbar using a home‐built 4‐pocket Knudsen‐type evaporator.

## Conflict of interest

The authors declare no conflict of interest.

## Supporting information

As a service to our authors and readers, this journal provides supporting information supplied by the authors. Such materials are peer reviewed and may be re‐organized for online delivery, but are not copy‐edited or typeset. Technical support issues arising from supporting information (other than missing files) should be addressed to the authors.

Supporting Information
