# Supplementary material for: Structure Formation and Coupling Reactions of Hexaphenylbenzene and Its Brominated Analog
Source: Chemphyschem. 2021 Jul 18;22(17):1769–73. doi: 10.1002/cphc.202100049 (PMC8456788; doi:10.1002/cphc.202100049)
Supplement: Supplementary file 1 — Supporting Information [file CPHC-22-1769-s001.pdf]

ChemPhysChem

Supporting Information

Structure Formation and Coupling Reactions of Hexaphenylbenzene and Its Brominated Analog

Jacob D. Teeter,* Paulo S. Costa, Christoph Dobner, Mamun Sarker, Alexander Sinitskii, and Axel Enders*

Supporting Information

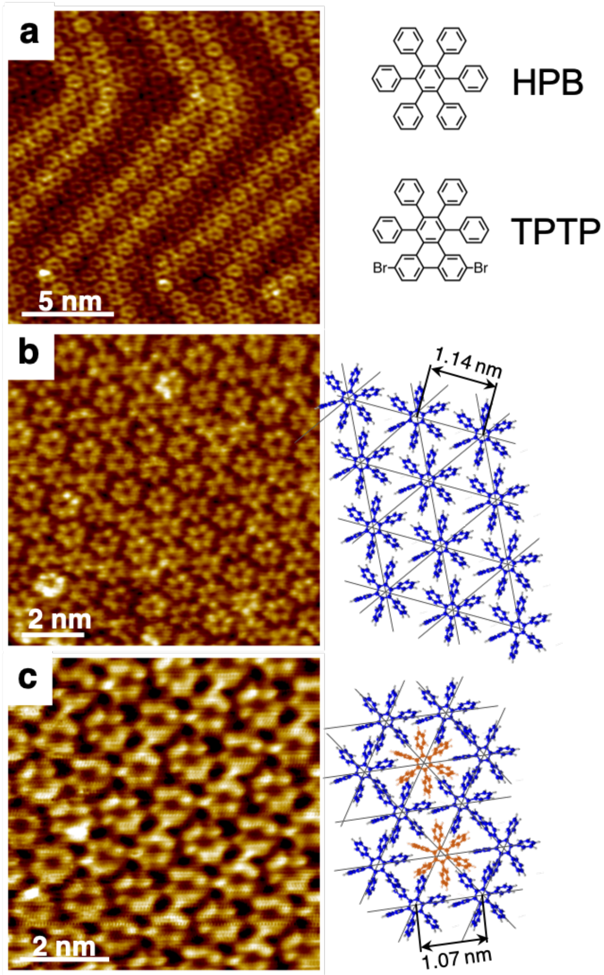

Figure 1: Copy of Figure 1 in the main article, but without superimposed structure models in the STM images.
